# Postmenopausal osteoporosis and vascular calcification: The estrogen regulation network and calcification paradox

**DOI:** 10.1515/jtim-2026-0026

**Published:** 2026-04-04

**Authors:** Yanxia Lin, Pengcheng Jiang, Yang Lv, Wen Tian

**Affiliations:** Department of Geriatrics, The First Hospital of China Medical University, Shenyang, Liaoning Province, China; Department of Infectious Diseases, The First Hospital of China Medical University, Shenyang, Liaoning Province, China

**Keywords:** postmenopausal, osteoporosis,vascular calcification, estrogen, estrogen receptor, epigenetic modification

## Abstract

The decline in estrogen levels in postmenopausal women is a major risk factor for the high prevalence of osteoporosis and vascular calcification in this population. Estrogen deficiency leads to an imbalance in the RANKL/OPG ratio, abnormalities in the Wnt/β-catenin pathway, and disruptions in bone morphogenetic protein (BMP) signaling, thereby inducing osteoporosis and vascular calcification. Moreover, estrogen deficiency affects calcium and phosphorus metabolism and induces the release of extracellular vesicles from the aging bone matrix, which mediate interactions between bone and vascular tissues and promote the occurrence of the "calcification paradox." The epigenetic regulation of estrogen receptors, including DNA methylation, histone modification, and miRNA activity, exerts different effects in bone and vascular tissues, underscoring the complexity of estrogen regulation. Future research should focus on the precise regulation of estrogen receptor subtypes and tissue-specific epigenetic modulation mechanisms to develop effective therapeutic strategies for osteoporosis and vascular calcification in postmenopausal women.

## Introduction

Osteoporosis and vascular calcification are common age-related conditions that increase the risk of falls, fractures, and adverse cardiovascular events in the elderly. During the 5 to 10 years following menopause, bone loss is most pronounced. According to a nationwide, multicenter survey of osteoporosis in China, the age-standardized prevalence of osteoporosis is 29.13% among women aged 50 years and older and 6.46% among men of the same age group.^[1]^ Meanwhile, the risk of cardiovascular disease significantly increases in postmenopausal women, who exhibit higher coronary artery calcification scores compared with premenopausal women. Studies have also demonstrated an association between arterial calcification and osteoporosis in postmenopausal women, a phenomenon known as the “calcification paradox,” in which the same individual experiences insufficient bone formation (osteoporosis) and excessive calcification of vascular walls (vascular calcification).

The decline in estrogen levels in postmenopausal women is widely recognized as a major risk factor for the increased incidence of osteoporosis and vascular calcification.^[2,3]^ Estrogen plays a crucial role in maintaining both bone density and cardiovascular health. When estrogen levels decline, bone resorption increases while bone formation decreases, leading to osteoporosis. Additionally, reduced estrogen levels are associated with dysfunction of vascular endothelial cells (VECs) and vascular smooth muscle cells (VSMCs), as well as the osteoblastic phenotypic transformation of VSMCs, which in turn promotes vascular calcification. Furthermore, as estrogen levels fall during the perimenopausal period, the inhibitory effect on the hypothalamus and pituitary gland weakens, resulting in increased synthesis of follicle-stimulating hormone (FSH).

Although osteoporosis and vascular calcification exhibit distinct histopathological characteristics, they share numerous overlapping mechanisms. Calcification of VSMCs and osteogenesis of bone tissue are regulated by many common signaling pathways, with key factors closely linked to estrogen action.^[4]^ Estrogen replacement therapy was once considered a treatment for osteoporosis and vascular calcification in postmenopausal women,^[3,5]^ but its clinical use has been limited due to tumorigenic risks.^[6]^

Estrogen regulates the expression of multiple genes through estrogen receptors (ERs), influencing bone metabolism and VSMC function, and modulating calcium deposition and bone remodeling. As transcription factors, activated ERs inhibit osteoblast apoptosis, promote skeletal health, and suppress vascular calcification by regulating VSMC phenotypic transformation. Therefore, elucidating the mechanisms through which estrogen regulates osteogenesis and vascular calcification is essential for developing comprehensive therapeutic strategies for postmenopausal women, providing a scientific foundation for health management in this population.

## Biological effects and mechanisms mediated by estrogen and its receptors

Estrogen exists in three major forms: estrone (E1), estradiol (E2), and estriol (E3). Before menopause, E2 is the predominant form, secreted primarily by the ovaries. After menopause, E1 becomes the principal form, produced by aromatase-mediated conversion of androstenedione (A2) in peripheral tissues, with small amounts of E2 originating from the adrenal cortex or converted from E1. The decline in estrogen levels leads to abnormal ER signaling, which is closely associated with osteoporosis, vascular calcification, impaired glucose and lipid metabolism, and breast cancer.

Two classes of receptors mediate estrogen action: the steroid hormone nuclear receptor superfamily (ERs) and the G protein-coupled estrogen receptor (GPER) superfamily. These receptor families mediate distinct modes of estrogen activity: genomic effects and non-genomic effects (rapid signaling).^[7]^ The genomic effects of estrogen involve ER-mediated transcriptional regulation, requiring several hours to days to exert their influence. Non-genomic effects are rapid, occurring within seconds to minutes, without direct involvement in transcriptional regulation.

The steroid nuclear receptor superfamily comprises two main subtypes, ERα and ERβ, each consisting of the following functional domains: the NH^2^-terminal domain (NTD), the DNA-binding domain (DBD), and the COOH-terminal ligand-binding domain (LBD). The NTD includes the ligand-independent activation function (AF1) domain, which participates in the transcriptional activation of target genes; ERα and ERβ share only 16% sequence similarity in this region. The DBD mediates the binding of ERs to estrogen response elements (EREs) in the promoter regions of target genes and is highly conserved, with ERα and ERβ sharing 97% amino acid identity. The LBD contains the ligand-dependent activation function (AF2) domain, with ERα and ERβ sharing nearly 60% overall sequence similarity. Despite this, the ligand-binding pockets of the two subtypes exhibit only minor structural differences. Under physiological conditions, ERs are primarily located in the cell nucleus but are also present in the cytoplasm and mitochondria. The structural differences between ERα and ERβ underlie their functional differences.^[8]^

ERα is mainly expressed in the breast, uterus, ovaries (principal cells), bone, male reproductive organs (testes and epididymis), prostate (stromal cells), liver, and adipose tissue, whereas ERβ is highly expressed in the prostate (epithelial cells), bladder, ovaries (granulosa cells), colon, adipose tissue, and immune system.^[9]^ In VSMCs, both ERα and ERβ are expressed at comparable levels, and inhibition of either receptor promotes the progression of vascular calcification.^[10]^ However, in vascular biology, ERα has been more strongly implicated in vascular injury and repair.

After binding and activation by a ligand, ERs regulate biological processes through several pathways. In the classical signaling pathway, ER dimers translocate into the nucleus upon ligand activation and directly bind to EREs in the promoter regions of target genes to regulate transcription. ERs may also interact with other transcription factors, such as AP-1 and Sp-1, through transcription factor crosstalk. In addition, ERs can be activated in the absence of a ligand through kinase-mediated phosphorylation. For instance, ERK1 and ERK2, signaling molecules downstream of HER2, can phosphorylate ER, resulting in ligand-independent activation.

Estrogen can also mediate rapid biological effects through the GPER, which is located on the plasma membrane or subcellular organelle membranes.^[11]^ These effects involve the participation of cytoplasmic signal transduction proteins. Confirmed signaling pathways include the stimulation of endothelial nitric oxide synthase (eNOS) and nitric oxide (NO) synthesis,^[12]^ regulation of the mitogen-activated protein kinase (MAPK) pathway, activation of adenylate cyclase and cAMP formation,^[13]^ subsequent activation of protein kinase A (PKA) and protein kinase C (PKC),^[14,15]^ and an increase in intracellular Ca2+ concentration.^[16]^

## Extragonadal biological effects of FSH and its mediated pathophysiological changes

Under physiological conditions, estrogen regulates the synthesis and secretion of FSH through negative feedback. FSH maintains the physiological balance between E2 and FSH by stimulating follicular development, maturation, and estrogen production. Before menopause, E2 levels begin to decline in a fluctuating manner. Due to the weakening of negative feedback inhibition, FSH levels increase markedly, typically preceding the decline in estrogen. Elevated FSH is considered the earliest serum marker of decreased ovarian reserve. After menopause, ovarian function ceases, E2 levels remain low, and FSH levels remain persistently high, often reaching more than ten times the levels observed during reproductive age.

FSH binds to its receptor (FSHR, a G protein-coupled receptor) to activate downstream signaling pathways and exert effects on target cells. It was previously believed that FSH exerted gonad-specific effects. However, recent studies have demonstrated that FSHR is widely expressed in extragonadal tissues, including osteoclasts, monocytes, VSMCs, and vascular endothelial cells.^[17]^

It is currently recognized that FSH influences bone metabolism independently of estrogen. Elevated FSH levels may contribute to bone loss during the perimenopausal period by promoting bone resorption. Studies have shown that FSH promotes the differentiation and functional activation of osteoclasts by activating the MEK/ERK, nuclear factor-κB (NF-κB), and Akt signaling pathways.^[18]^ The same research group demonstrated that mice deficient in the FSH β-subunit are protected from bone loss despite severe estrogen deficiency. FSH has also been shown to stimulate bone marrow granulocytes and macrophages to produce tumor necrosis factor-alpha (TNF-α), thereby expanding the population of osteoclast precursor cells.^[19]^

FSH can directly accelerate atherosclerosis in ovariectomized ApoE–/– mice by activating the cAMP/PKA and PI3K/Akt/mTOR/NF-κB signaling pathways in vascular endothelial cells. This activation upregulates vascular cell adhesion molecule-1 (VCAM-1) expression, enhances monocyte adhesion, and occurs independently of estrogen deficiency.^[20]^ Moreover, FSH can promote angiogenesis by activating the PI3K/Akt signaling pathway in human umbilical vein endothelial cells (HUVECs), independently of vascular endothelial growth factor (VEGF).^[21]^ Functional FSHR expression has also been detected in adipocytes. In mouse preadipocytes, FSH promotes lipid biosynthesis and lipid droplet formation through the Ca^2+^/cAMP regulatory element-binding protein (CREB) pathway.^[22]^ Furthermore, FSH can increase circulating low-density lipoprotein cholesterol (LDL-C) levels by reducing LDL receptor expression in hepatocytes.^[23]^

## Molecular mechanisms of estrogen in regulating bone metabolism and its impact on osteoporosis

Under physiological conditions, bones undergo continuous self-renewal to preserve mechanical strength. The dynamic process of bone metabolism consists of bone resorption, dominated by osteoclasts, and bone formation, dominated by osteoblasts, encompassing five stages: activation, resorption, reversal, formation, and mineralization. When bone cells sense mechanical stress, they release signaling molecules such as receptor activator of NF-κB ligand (RANKL), which recruit osteoclast precursors. Lining cells are separated to expose the bone surface, initiating the resorption phase. During this phase, osteoclasts dissolve bone minerals and collagen matrix by secreting hydrogen ions and lysosomal enzymes, thereby forming resorption cavities and releasing calcium and phosphate into the circulation. Subsequently, the reversal phase begins, during which osteoclasts undergo apoptosis and osteoblast precursors are recruited to the resorption cavity. Osteoblasts then secrete type I collagen and osteocalcin, forming osteoid tissue, which undergoes mineralization. Osteoblasts subsequently differentiate into osteocytes or undergo apoptosis. During this period, the new bone matrix matures and solidifies. This cycle typically lasts three to six months, with approximately 10% of the adult skeleton being renewed annually.

Bone metabolism is primarily regulated by the receptor activator of NF-κB (RANK)/RANK Ligand (RANKL)/osteoprotegerin (OPG) pathway and the canonical Wnt/β-catenin signaling pathway. It is further modulated by endocrine factors (parathyroid hormone, vitamin D, sex hormones, calcitonin, growth hormone, and glucocorticoids) and paracrine factors (cytokines and growth factors).

RANKL, secreted by osteoblasts, binds to RANK receptors on osteoclast precursors, thereby activating the NF-κB and MAPK pathways to promote osteoclast differentiation and survival. OPG acts as a decoy receptor for RANKL, inhibiting RANK–RANKL interactions and reducing osteoclastogenesis. Activation of the BMP/Smad or Wnt/β-catenin pathways in osteoblasts induces the nuclear translocation of Smad or β-catenin proteins, where they function as transcription factors or co-regulators to upregulate osteogenesis-related genes such as Runx2, thereby promoting bone formation. Sclerostin (SOST) and Dickkopf-1 (DKK1), natural inhibitors of Wnt signaling secreted by osteocytes, competitively bind to the Wnt co-receptor LRP5/6, block Wnt–β-catenin signaling, and inhibit bone formation through negative feedback. Under physiological conditions, mechanical stimulation suppresses sclerostin expression, and estrogen also downregulates SOST and DKK1 expression, thereby alleviating Wnt pathway inhibition and enhancing bone formation.^[24]^

Following menopause, the marked decline in estrogen levels results in the uncoupling of bone resorption and bone formation. In this state, osteoblast-mediated bone formation cannot compensate for the increased osteoclast-mediated bone resorption, ultimately leading to bone loss and osteoporosis.

Estrogen inhibits the synthesis and secretion of RANKL and upregulates OPG expression, thereby maintaining the balance between bone resorption and formation. It can also activate the Wnt/β-catenin signaling pathway, promoting osteoblast differentiation and bone formation.^[25]^ Estrogen exerts antioxidant effects by reducing reactive oxygen species (ROS) levels and inducing the expression of mitochondrial respiratory chain complexes, thereby enhancing osteoblast energy metabolism.^[26]^ Furthermore, estrogen upregulates FasL through ERα, accelerating osteoclast apoptosis mediated by the Fas/FasL system.^[27]^ In addition to directly participating in bone metabolism, estrogen indirectly regulates bone metabolism by influencing calcium and phosphorus metabolism. It upregulates intestinal calcium channel expression, increases calcium absorption, and acts synergistically with vitamin D to enhance renal 1α-hydroxylase activity, thereby raising active vitamin D levels and promoting bone mineralization.^[28]^ When estrogen levels decline significantly, bone marrow mesenchymal stem cells tend to differentiate into adipocytes rather than osteoblasts, whereas estrogen intervention can reverse this trend.^[29]^ In addition, ROS can impair mitochondrial function in osteoblasts while simultaneously promoting osteoclast differentiation. A reduction in estrogen levels affects bone metabolism through multiple mechanisms, resulting in decreased bone density or osteoporosis and an increased risk of fractures.

## Regulation of VECs and VSMCs function by estrogen and its receptors and their impact on vascular calcification

Estrogen regulates the biological functions of VECs and VSMCs, including vascular contraction and relaxation, cell proliferation and migration, and phenotypic transformation, through genomic effects mediated by ERs and non-genomic effects mediated by GPER.

Estrogen mitigates vascular calcification by suppressing RANKL and BMP2 expression in VECs, an effect mediated primarily *via* estrogen receptor α (ERα).^[4]^ ERα-dependent estrogen signaling further stimulates the release of vasoactive mediators including NO and prostacyclin from VECs. Beyond these direct effects, estrogen exerts vascular protective actions through anti-inflammatory activity and epigenetic modulation, and reshapes the endothelial transcriptomic landscape with microRNAs (miRNAs) implicated in vascular regulatory cascades.^[30]^ Profiling of miRNA expression coupled with microarray validation following E2 treatment revealed that miR-30b-5p, miR-487a-5p, miR-4710 and miR-501–3p were upregulated, whereas miR-378 h and miR-1244 were downregulated in E2-exposed VECs. The ER-dependent, E2-regulated differentially expressed miRNA pathways in human endothelial cells indicate that miRNAs may act as key epigenetic regulators of gene expression in E2-treated endothelial cells.^[31]^

In VSMCs, Estrogen downregulates the expression of Cav1.2 channels (L-type voltage-gated calcium channels) through ERα, thereby reducing Ca^2+^ influx and inhibiting vascular contraction.^[32]^ It can also rapidly inhibit L-type calcium channels in a non-genomic manner, further reducing Ca^2+^ influx.^[33]^ ERα-mediated nuclear action is both necessary and sufficient to inhibit postinjury arterial VSMC proliferation.^[34]^ E2 downregulates NF-κB expression to suppress inflammation-mediated VSMC proliferation.^[35]^ E2 interacts with ERα and liver X receptor α (LXRα) to upregulate ABCA1 expression, markedly enhancing cholesterol efflux to high density lipoprotein (HDL), thereby reducing cholesterol ester accumulation in VSMCs and macrophages and decreasing foam cell formation.^[36,37]^ ERβ activation selectively antagonizes TNF-α signaling, reducing the release of chemokines such as IL-6 and MCP-1, and lowering plaque inflammation.^[38]^ Estrogen also inhibits VSMC proliferation and migration through the MAPK/ERK, Ras-pRb, and PI3K/Akt pathways, thereby delaying the formation and progression of atherosclerotic plaques.^[39]^ ERα activation promotes VSMC autophagy, decreases the expression of RUNX2 and ALP, and consequently inhibits osteoblastic differentiation and vascular calcification in VSMCs.^[40]^ GPER activation inhibits VSMC migration and phenotypic transformation through the ERK1/2 and PI3K/Akt pathways and synergizes with ERα to enhance vascular protection by inhibiting calcium influx and promoting vasodilation.^[39,41]^

Vascular calcification is a complex, dynamic process. Under conditions such as abnormal calcium and phosphorus metabolism, oxidative stress, dysregulated glucose metabolism, DNA damage, abnormal epigenetic modifications, and aging, the osteoblastic phenotypic transformation of VSMCs plays a central role. This process is characterized by decreased expression of VSMC contractile markers (*e.g*., α-SMA and SM-22α) and increased expression of osteoblast markers (*e.g*., Runx2 and BMP2). It is frequently accompanied by elevated tissue non-specific alkaline phosphatase and hydroxyapatite-rich matrix vesicles, along with reduced levels of calcification protective factors such as fetuin and matrix Gla protein.^[42]^

Studies have demonstrated that estrogen plays a critical protective role in maintaining vascular health and inhibiting vascular calcification. Estrogen inhibits vascular calcification in an ERα-dependent manner, while estrogen deficiency upregulates calcification-related proteins and promotes calcium deposition in the aorta.^[4]^ Estrogen also suppresses arterial calcification by enhancing autophagy, and selective ERα antagonists MPP and ERα-specific siRNA abolish the inhibitory effects of estrogen on arterial calcification and estrogen-induced autophagy.^[40]^ ERα activation can reverse the downregulation of growth arrest-specific 6 (Gas6) in phosphorus-induced vascular calcification, thereby alleviating vascular calcification.^[43]^ Puerarin, a phytoestrogen, significantly reduces alkaline phosphatase activity, osteocalcin secretion, and Runx2 expression in VSMCs through the ER/PI3K/Akt signaling pathway in a dose-dependent manner, inhibits osteoblastic differentiation of VSMCs, and decreases vascular calcification nodules and calcium deposition.^[44]^

In clinical trials of osteoporosis treatment in postmenopausal women with a history of atherosclerosis, treatment with the selective ER modulator (SERM) raloxifene reduced cardiovascular events.^[45]^ In animal models of atherosclerosis in postmenopausal females, SERM reduced the expression of cyclooxygenase-2, matrix metalloproteinase- 1, monocyte chemoattractant protein-1, and macrophage infiltration in lesions, indicating anti-inflammatory effects, which were accompanied by upregulation of ERα and reduced vascular calcification.^[46]^ Although SERMs exhibit different therapeutic effects in breast and bone tissues, and do not increase the carcinogenic risk associated with estrogen replacement therapy, they may still increase the risk of fatal stroke and venous thromboembolism.^[47]^ These findings suggest the need for the development of tissue- or cell-specific ER regulators, such as agents that act exclusively on the LBD (AF2) domain of ERs in target cells, thereby producing specific effects in target cells without affecting other tissues.

## Common roles of estrogen and FSH in osteoporosis and vascular calcification and the molecular mechanisms of the “calcification paradox”

**Figure 1:**
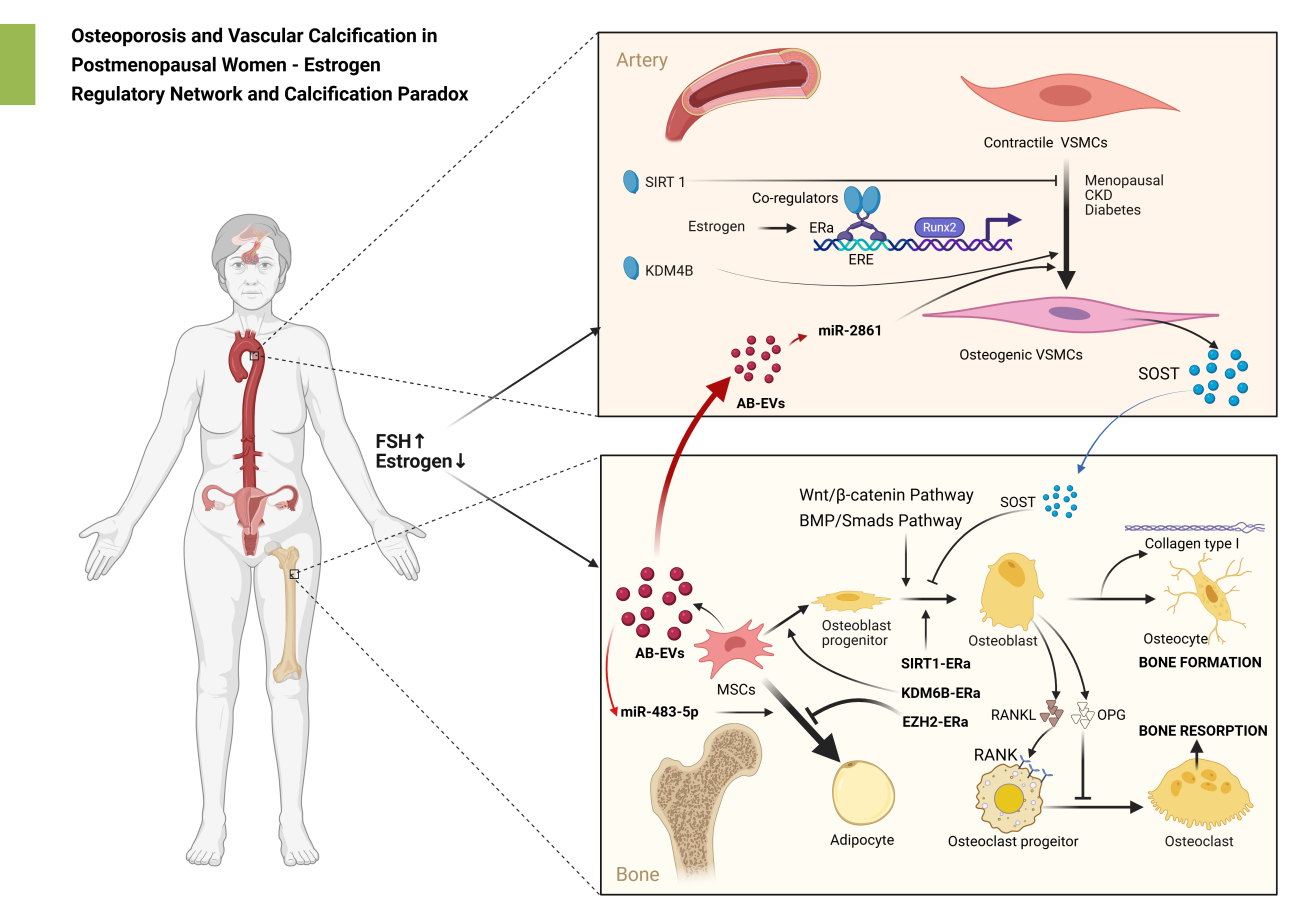
The mechanisms of osteoporosis and vascular calcification in postmenopausal women - estrogen regulatory network and calcification paradox. AB-EVs: EVs released from aging bone matrix; BMP: bone morphogenetic proteins; CKD: chronic kidney disease; ER: estrogen receptor; ERE: estrogen response element; EZH2: enhancer of Zeste Homolog 2; FSH: follicle stimulating hormone; Gas 6: growth arrest-specific gene 6; H3K27me3: trimethylated histone H3 at lysine 27; KDM: histone lysine demethylase; MSC: mesenchymal stem cells; PPARγ: peroxisome proliferator-activated receptors gamma; PRC2: polycomb repressive complex 2; RANK: NF-κB receptor activator; RANKL: RANK ligand; OPG: osteoprotegerin; Runx2: runt-associated transcription factor 2; SIRT1: Sirtuin 1; SOST: sclerostin; VSMCs: vascular smooth muscle cells. Created with BioRender.com.

Estrogen and its receptors play a central role in bone metabolism and vascular homeostasis through diverse molecular mechanisms. The decline in estrogen levels and dysfunction of ERs may represent a key pathological link connecting osteoporosis (OP) and vascular calcification (VC) (Figure 1).

RANKL/RANK/OPG signaling axis: Estrogen regulates the balance of the OPG/RANKL ratio by inhibiting RANKL expression in osteoblasts and promoting OPG secretion. A reduction in estrogen levels decreases the OPG/RANKL ratio, leading to enhanced osteoclast differentiation and increased bone resorption. In the vascular system, estrogen deficiency-induced upregulation of RANKL expression triggers the osteoblastic phenotypic transformation of VSMCs through the BMP2 signaling cascade, thereby promoting hydroxyapatite deposition. Estrogen replacement therapy increases OPG levels while inhibiting RANKL expression, providing dual protection for the bone–vascular system.^[4]^

Wnt/β-catenin signaling pathway: Activation of the Wnt signaling pathway promotes osteoblast differentiation in bone tissue, and estrogen enhances both the early and late differentiation of bone marrow mesenchymal stem cells into osteoblasts through ERα activation, thereby facilitating bone formation.^[48]^ However, in the vascular system, abnormal activation of the non-canonical Wnt signaling pathway induces vascular calcification.^[49]^ Reports have also indicated that Wnt1 can inhibit vascular calcification in chronic kidney disease through the Wnt/β-catenin signaling pathway.^[50]^ To date, no studies have reported the involvement of estrogen in Wnt pathway-mediated vascular calcification. Previous investigations have demonstrated that the expression of SOST in calcified VSMCs is upregulated under high-phosphorus induction. In patients with end-stage renal disease or diabetes, plasma SOST concentrations are elevated and positively correlated with the severity of vascular calcification.^[51]^ This finding suggests that a negative feedback mechanism may exist, in which increased local synthesis of SOST in calcified arteries limits further vascular calcification. Elevated plasma SOST levels may inhibit the Wnt/β-catenin signaling pathway, thereby impairing osteogenesis and contributing to the crosstalk between vascular lesions and bone demineralization.

Bone morphogenetic protein (BMP) signaling pathway: BMPs, members of the transforming growth factor-β (TGF-β) superfamily, are critical regulators of osteoblast differentiation. Estrogen has been shown to upregulate BMP-6 protein expression in osteoblasts, and this effect can be abolished by estrogen receptor antagonists, implicating BMP-6 as a potential downstream mediator of estrogen-induced osteogenesis.^[52]^ Following menopause, declining estrogen levels lead to reduced BMP pathway activity in bone, while simultaneously enhancing RANKL-mediated signaling, which promotes osteoclast differentiation and activity. This shift accelerates bone resorption and contributes to the pathogenesis of osteoporosis. In VSMCs, estrogen deficiency results in elevated RANKL expression and downregulation of matrix Gla protein (MGP), a calcification inhibitor. RANKL secreted by VSMCs stimulates BMP-2 production by vascular endothelial cells, which in turn acts in a paracrine manner to promote osteogenic trans-differentiation of VSMCs. Under physiological conditions, MGP binds to and neutralizes BMP-2, thereby inhibiting its pro-calcific effects. However, reduced MGP expression in estrogen-deficient states diminishes this inhibitory control, indirectly potentiating BMP-2-mediated vascular calcification. Estrogen supplementation has been shown to counteract this process by suppressing RANKL-BMP2 signaling and mitigating calcification.^[4]^

Estrogen receptor (ER) gene polymorphisms: Mutations or polymorphisms in ER genes exert important effects on bone metabolism as well as on vascular structure and function. The rs2234693: T > C (PvuII) polymorphism is a common restriction fragment length polymorphism located in the first intron of the ERα gene. The PP genotype represents the absence of the restriction site for the PvuII restriction enzyme, pp denotes the presence of the site, and Pp indicates heterozygosity. The pp genotype is generally associated with a reduced response to estrogen stimulation compared with the PP or Pp genotypes and is linked to postmenopausal bone loss. Women carrying the PP genotype exhibit significantly higher bone mineral density (BMD) compared with those carrying the pp genotype.^[53]^ An autopsy study further confirmed that, in male patients aged 53 years and older, the calcified area of coronary atherosclerotic plaques in those with the PP genotype was approximately twice that observed in patients with the Pp or pp genotypes.^[54]^ These findings suggest that ERα plays a crucial role in the concurrent processes of bone metabolism and atherosclerotic calcification.

Calcium and phosphorus metabolism disorders: Disturbances in calcium and phosphorus metabolism constitute the material basis for the “calcification paradox”. Estrogen deficiency leads to reduced intestinal calcium absorption, increased renal calcium excretion, and enhanced release of calcium and phosphorus from bone. These metabolic alterations ultimately elevate blood calcium and phosphorus concentrations, promoting the deposition of hydroxyapatite in the vascular wall. Furthermore, inter-organ communication mediated by extracellular vesicles (EVs) released from the aging bone matrix provides a theoretical explanation for the “calcification paradox”.^[55]^ Studies have demonstrated that EVs derived from aging bone matrix (AB-EVs) transport regulatory molecules such as miR-483–5p and miR-2861. miR-483–5p enhances PPARγ expression in bone marrow mesenchymal stem cells, inducing adipogenic differentiation and suppressing osteogenic differentiation. AB-EVs also enter the circulation and deposit in the vascular wall, where they stimulate RUNX2 expression in VSMCs, promoting osteoblastic transformation and vascular calcification. Estrogen deficiency significantly increases the release of AB-EVs, thereby accelerating bone loss and inducing vascular calcification. This evidence reveals a novel “bone–vascular axis” regulatory mechanism in which AB-EVs simultaneously regulate bone metabolism and influence vascular calcification across organs. Moreover, treatment with the bone resorption inhibitor alendronate reduces AB-EV release, alleviating both osteoporosis and vascular calcification in the elderly. This observation aligns with previous preclinical studies suggesting that bisphosphonates inhibit vascular calcification while suppressing bone resorption.^[56,57]^ A small clinical study of 35 dialysis patients with a mean age of 63 years demonstrated that etidronate effectively reduced coronary artery calcification scores.^[58]^ However, most subsequent clinical trials failed to confirm that bisphosphonates or other bone resorption inhibitors effectively reduce aortic or coronary artery calcification in elderly patients with osteoporosis.^[59, 60, 61]^ These discrepancies may be attributed to the older age of participants, advanced disease stages, and diverse clinical backgrounds. By contrast, preclinical animal studies are typically conducted in early disease stages without confounding factors or comorbidities.

The markedly elevated FSH levels observed in perimenopausal women may represent a key mechanism underlying the “calcification paradox”. FSH directly binds to FSHR on osteoclast precursor cells and upregulates the nuclear factor of activated T cells cytoplasmic 1 (NFATc1) *via* the cAMP/PKA/CREB pathway, thereby promoting osteoclast differentiation. Animal studies demonstrate that elevated FSH levels increase the number of osteoclasts and reduce bone mineral density. FSH also stimulates immune cells to release TNF-α, activates RANKL signaling, and amplifies bone resorption. Additionally, FSH inhibits osteoblast differentiation by upregulating DKK1 and reduces OPG expression, resulting in an increased RANKL/OPG ratio and accelerated bone loss. Treatment with anti-FSHR antibodies enhances bone mineral density and increases trabecular bone number in ovariectomized rats.

Furthermore, FSH upregulates Runx2, a critical transcription factor for osteogenesis, through the FSHR/Gαs/cAMP/PKA pathway, thereby promoting the transdifferentiation of VSMCs into osteoblast-like cells. Under high-phosphorus conditions, FSH increases the formation of calcium nodules and alkaline phosphatase activity in VSMCs. FSH also activates the PI3K/Akt/mTOR/NF-κB pathway, upregulates VCAM-1 expression, promotes monocyte adhesion and inflammatory factor release, and accelerates vascular calcification. In the postmenopausal state, the combination of elevated FSH and low estrogen enhances bone calcium release and promotes ectopic vascular calcium deposition.

## Epigenetic regulation of ERs and its implications for the “calcification paradox”

As a transcription factor, ER activity is dynamically regulated by various epigenetic modifications, including DNA methylation, histone methylation/demethylation, histone acetylation/deacetylation, and non-coding RNA regulation. These modifications alter chromatin accessibility, co-regulator recruitment, and DNA–protein interactions, thereby profoundly influencing ER-mediated biological effects. While epigenetic regulation of ERs has been widely studied in cancer research, it has rarely been investigated in relation to bone metabolism and vascular calcification.

The ER exhibits opposite regulatory roles in bone and vascular tissues. In bone tissue, ERα promotes osteoblast differentiation and bone formation, whereas in vascular tissue, it suppresses osteoblastic differentiation of VSMCs and vascular calcification. This contradictory regulation may be attributable to differences in co-regulator interactions and epigenetic modifications. Distinct co-regulators may bind to ER in different cell types, leading to divergent transcriptional outcomes. Such complex interactions determine the multifunctional roles of estrogen in diverse tissues, providing new insights into its biological actions and offering potential therapeutic targets for osteoporosis and vascular calcification.

Methylation of CpG islands in the ERα promoter region is directly associated with gene silencing. Studies have shown that the methylation level of the ERα promoter in osteoblasts of postmenopausal osteoporosis patients is significantly elevated, resulting in reduced ERα expression.^[62]^ When estrogen levels decline, bone marrow mesenchymal stem cells (MSCs) preferentially differentiate into adipocytes rather than osteoblasts. Estrogen intervention activates ERα to recruit EZH^2^, the core subunit of polycomb repressive complex 2 (PRC2) with histone methyltransferase activity, to the promoters of adipocyte differentiation-related genes such as PPARγ. This recruitment leads to H3K27 methylation, producing the repressive transcriptional markers H3K27me2 and H3K27me3, thereby suppressing transcription of these genes and inhibiting adipogenic differentiation of MSCs.^[29]^ In addition, ERα can be recruited to the promoter of lysine-specific demethylase 6B (KDM6B) following estrogen stimulation, upregulating KDM6B expression. KDM6B subsequently binds to the BMP2 promoter, removes the H3K27me3 mark, and activates BMP2 transcription. Inhibition of KDM6B markedly reduces the osteogenic potential of MSCs, suggesting that the ERα/KDM6B axis is essential in the epigenetic regulation of estrogen-dependent osteogenesis.^[63]^

Our recent findings demonstrated that KDM4B is highly expressed in β-glycerophosphate-induced calcified VSMCs and suppresses ERα-induced transcriptional activity. The inhibitory effect of KDM4B on ERα-driven transcription does not depend on its demethylase activity but instead involves recruitment of the PRC2 complex to the estrogen response element (ERE) of the ERα target gene Gas6, thereby increasing H3K27me3 levels. Knockdown of KDM4B reduces PRC2 recruitment, decreases H3K27me3 enrichment, and enhances mRNA expression of endogenous ERα target genes. Estrogen intervention attenuates the pro-calcific effect of KDM4B on VSMCs. These findings indicate that KDM4B functions as a corepressor of ERα in the regulation of vascular calcification and may represent a novel therapeutic target for vascular calcification.

The histone deacetylase Sirtuin 1 (SIRT1) also plays an important role in osteogenesis. Studies have confirmed that estrogen promotes MSC differentiation toward osteoblasts and enhances osteoblast function by upregulating SIRT1.^[64,65]^ SIRT1 is recognized as a positive regulator of Runx2, a master transcription factor of osteogenesis.^[66]^ Conversely, SIRT1 downregulation plays a central role in postmenopausal vascular calcification. The phytoestrogen resveratrol alleviates arterial calcification in ovariectomized rats by activating the SIRT1 pathway and downregulating Runx2 as well as the senescence markers p16 and p21.^[67]^ A recent study further demonstrated that nicotinamide phosphoribosyl transferase (NAMPT) inhibits vascular calcification by increasing NAD+ synthesis and activating SIRT1.^[68]^ It was demonstrated that estrogen may suppress histone H3 acetylation by activating SIRT1,^[69]^ whereas H3 acetylation is widely recognized as a stimulatory marker of gene transcription. The H3 acetylation typically occurs on lysine residues, such as H3K9ac, H3K27ac, *etc*., which neutralizes the positive charge of lysine, weakening the interaction between histones and DNA, thus making chromatin more loose (open state) and promoting the binding of transcription factors and RNA polymerase to DNA.

Collectively, these studies suggest that ERs exert cell-specific effects through differential recruitment of co-regulators, such as KDM6B in osteoblasts and KDM4B in VSMCs, thereby regulating transcription of distinct target genes. At the same time, shared co-regulators of ERs, such as SIRT1, are expressed in MSCs, osteoblasts, and VSMCs but mediate divergent outcomes depending on the cellular context. These findings imply the existence of a highly complex transcriptional regulatory network that varies across different tissues and cell types (Table 1). The interaction of ERs, co-regulators, and target genes is influenced by promoter sequences, cell-specific features, and microenvironmental factors, and the consequences of these interactions may differ significantly among tissues. However, the precise mechanisms remain to be elucidated.

**Table 1:** Co-regulators of ERa for epigenetic mechanisms in osteoporosis and vascular calcification

Cell type	Co-regulators	Target genes	Effects
MSCs	EZH_2_	PPARγ	Upregulate H3K27me3, inhibiting the transcription of PPARγ, suppressing the differentiation of MSCs into adipocytes
	KDM6B	BMP2	Remove the H3K27me3 and activating BMP2 transcription, promote the osteogenic differentiation potential of MSCs
	SIRT1	Not mentioned	promote the osteogenic differentiation potential of MSCs
Osteoblast	SIRT1	Runx2	the positive regulator of the osteoblast main transcription factor-Runx2
VSMCs	KDM4B	Gas 6	recruit PRC2 complex to promoter ERE regions of Gas6 gene, up-regulate the H3K27me3 levels and inhibit the transcription of Gas6 gene
	SIRT1	Runx2	down-regulate the expression of Runx2 and inhibit the osteogenic differentiation of VSMCs

BMP2: Bone morphogenetic proteins2; ERE: estrogen response element; EZH2: Enhancer of Zeste Homolog 2; Gas 6: growth arrest-specific gene 6; H3K27me3: trimethylated histone H3 at lysine 27; KDM: Histone lysine demethylase; MSC: msenchymal stem cells; PPARγ: peroxisome proliferator-activated receptors gamma; PRC2: polycomb repressive complex 2; Runx2: runt-associated transcription factor 2; SIRT1: Sirtuin 1; VSMCs: vascular smooth muscle cells;

miRNAs, as non-coding RNAs, regulate the expression of target genes at the post-transcriptional level by binding to specific gene sequences. Numerous studies have demonstrated that changes in miRNA expression in women are closely associated with estrogen. In patients with postmenopausal osteoporosis, miR-338 is highly expressed, which impairs osteoblast differentiation by downregulating genes that promote this process, such as Runx2. During osteogenic differentiation, estrogen-dependent Runx2 functions as a transcription factor to negatively regulate miR-338 expression. Estrogen thus regulates osteoblast differentiation through a positive and negative feedback loop composed of miR-338 and Runx2. Inhibition of miR-338 in the early stage after ovariectomy can effectively prevent and delay the progression of osteoporosis. These findings suggest that the miR-338 cluster may serve as a potential diagnostic marker and therapeutic target for postmenopausal osteoporosis.^[70]^ Estrogen also inhibits osteoclast differentiation while significantly increasing miR-27a transcription. MiR-27a binds to the 3′UTR of PPARγ, suppresses PPARγ expression in osteoclasts, and markedly enhances the inhibitory effect of estrogen on bone resorption. Conversely, downregulation of miR-27a attenuates the inhibitory effects of estrogen on osteoclast differentiation and bone resorption.^[71]^ Downregulation of miR-133b and miR-211, and upregulation of miR-29b, have been observed in uremic rats fed a high-phosphate diet, which correlated respectively with increased expression of the osteogenic transcription factor RUNX2. Furthermore, the use of antagomirs and mimics to modulate the expression levels of miR-29b, miR-133b, and miR-211 confirmed that these miRNAs regulate the calcification process.^[72]^ Many miRNAs have been identified to be associated with postmenopausal osteoporosis and vascular calcification, as summarized in a recently published review.^[73]^

## Conclusions and perspectives

**Figure 2:**
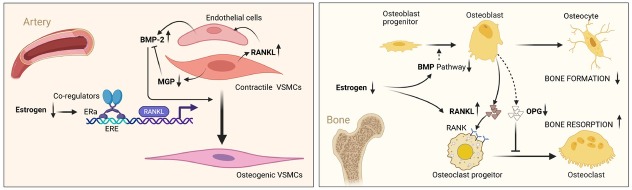
The effects of estrogen deficiency on vasculature and bone tissue - The mechanism of RANKL-BMP pathway. In VSMCs (left panel), estrogen deficiency results in elevated RANKL expression and downregulation of MGP, a calcification inhibitor. RANKL secreted by VSMCs stimulates BMP-2 production by vascular endothelial cells, which in turn acts in a paracrine manner to promote osteogenic trans-differentiation of VSMCs. Reduced MGP expression in estrogen-deficient states diminishes this inhibitory control, indirectly potentiating BMP-2-mediated vascular calcification. In bone tissue, declining estrogen levels lead to reduced BMP pathway activity in bone, while simultaneously enhancing RANKL-mediated signaling, which promotes osteoclast differentiation and activity. This shift accelerates bone resorption and contributes to the pathogenesis of osteoporosis. BMP: bone morphogenetic proteins; ER: estrogen receptor; MGP: matrix Gla protein; OPG: osteoprotegerin; RANKL: NF-κB receptor activator ligand; VSMC: vascular smooth muscle cell. Created with BioRender.com.

Postmenopausal osteoporosis and vascular calcification are closely interrelated, with estrogen deficiency serving as a common driving factor. Estrogen regulates not only bone metabolism but also vascular function, as well as the transdifferentiation and biological behavior of VSMCs. The ER-mediated gene transcriptional regulatory network displays paradoxical osteogenic mechanisms in bone and vascular tissues, which may be attributable to the tissue- and cell-specific actions of ERs, thereby revealing highly complex regulatory processes (Figure 2). Elucidating these mechanisms will identify potential diagnostic and therapeutic targets for both VC and OP in postmenopausal women. Additionally, accounting for the treatment responses of patients with distinct ER genotypes to estrogen or SERMs, and optimizing the management of comorbidities (*e.g*., diabetes mellitus, chronic kidney disease), are indispensable components of therapeutic strategies for postmenopausal OP and VC. The miRNA components of AB-EVs, such as miR-483–5p, may serve as biomarkers for the early detection of postmenopausal osteoporosis complicated by vascular calcification, thereby enabling early diagnosis, prevention, and treatment. Epigenetic regulatory factors may also contribute to the calcification process under estrogen-deficient conditions and represent potential therapeutic targets for both postmenopausal osteoporosis and vascular calcification. Future research should focus on the precise regulation of tissue- and cell-specific ER subtypes, the epigenetic transcriptional regulation of ERs, and the mutual interactions between postmenopausal osteoporosis and vascular calcification. Such investigations may ultimately provide safe and effective therapeutic strategies for affected patients.
